# Sodium antiporters of *Pseudomonas aeruginosa* in challenging conditions: effects on growth, biofilm formation, and swarming motility

**DOI:** 10.1186/s43141-020-0019-y

**Published:** 2020-02-03

**Authors:** Carla B. Schubiger, Kelli H. T. Hoang, Claudia C. Häse

**Affiliations:** 1 Department of Biomedical Sciences, Carlson College of Veterinary Medicine, Oregon State University, Corvallis, OR 97331 USA; 2 College of Pharmacy, Oregon State University, Corvallis, OR 97331 USA

**Keywords:** *Pseudomonas aeruginosa*, Sodium, Antiporter, pH, Growth, Motility

## Abstract

**Background:**

*Pseudomonas aeruginosa* is a bacterial pathogen that can cause grave and sometimes chronic infections in patients with weakened immune systems and cystic fibrosis. It is expected that sodium/proton transporters in the cellular membrane are crucial for the organism’s survival and growth under certain conditions, since many cellular processes rely on the maintenance of Na^+^ and H^+^ transmembrane gradients.

**Results:**

This study focused on the role of the primary and secondary proton and/or sodium pumps Mrp, Nuo, NhaB, NhaP, and NQR for growth, biofilm formation, and swarming motility in *P. aeruginosa*. Using mutants with gene deletions, we investigated the impact of each sodium pump’s absence on the overall growth, biofilm formation, motility, and weak acid tolerance of the organism. We found that the absence of some, but not all, of the sodium pumps have a deleterious effect on the different phenotypes of *P. aeruginosa*.

**Conclusion:**

The absence of the Mrp sodium/proton antiporter was clearly important in the organism’s ability to survive and function in environments of higher pH and sodium concentrations, while the absence of Complex I, which is encoded by the *nuo* genes, had some consistent impact on the organism’s growth regardless of the pH and sodium concentration of the environment.

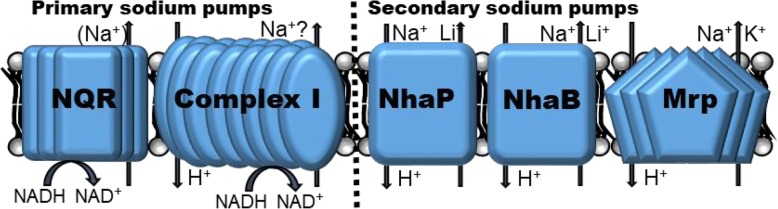

## Background

*Pseudomonas aeruginosa* is a ubiquitous opportunistic pathogen that can cause severe acute to chronic infections in humans. Among many other targets, it affects the skin, lungs, sensory organs, and urinary tract [15]. It is particularly devastating as a hospital-acquired infection and thrives in a variety of environments due to a large number of virulence factors and survival strategies. Besides the ability to quickly develop multidrug resistance, it also forms biofilms which make the bacteria more persistent and more resilient to treatment strategies [8, 11]. *P. aeruginosa* is one of the leading problems in hospital-acquired infections, cystic fibrosis, and immunocompromised patients [29]. In order to survive in these diverse environments, *P. aeruginosa* is equipped with a variety of cation transporters in the cellular membrane, which likely are essential to its physiology. Besides cation transport, some of the transporters import nutrients like amino acids and sugars, maintain the intracellular pH, and are key to flagellar movement, which enables *P. aeruginosa* to move quickly through liquid or soft material and across semi-solid surfaces in a swimming or swarming motion [39, 46].

The cation pumps investigated in this work are the Multiple Resistance and pH antiporter (Mrp, also called Sha), Complex I encoded by the *nuo* gene cluster, and the NADH-ubiquinone oxidoreductase NQR, as well as the sodium/proton antiporters NhaB and NhaP. NQR and Complex I are generally regarded as primary pumps that expel sodium or protons from the cytoplasm to the periplasm by directly using metabolic energy and are generally regarded crucial for the establishment of an electrochemical membrane cation gradient. NQR is a respiration-linked protein that couples NADH oxidation with sodium transport in many species [40], including *Vibrio cholerae* [3, 18], *V. alginolyticus* [4, 19], *V. harveyi* [51], *Haemophilus influenzae* [20], and *Klebsiella pneumonia* [6]. Similarly, Complex I couples NADH oxidation with proton transport and is confirmed to also transport sodium in *K. pneumoniae* and *Escherichia coli* [16, 44]. NQR and Complex I both contribute to the electron transport chain of *P. aeruginosa*, thus contributing to ATP generation and are possibly important to energy production and the maintenance of the electrochemical gradient [2, 50]. Just recently, however, Raba et al. [37] suggested that Pa-NQR is not a sodium pump, but acts as a H^+^-NQR. Therefore, our study will seek to confirm this new finding and will also investigate the sodium transporting involvement of *P. aeruginosa* Complex I.

Secondary sodium transporters utilize ΔpH, the electrochemical proton gradient, to expel sodium in exchange for protons [22]. Interestingly, the most intensely studied and widely present antiporter NhaA is missing in *P. aeruginosa*. However, it expresses NhaP and NhaB which are single subunit transporters [28]. NhaP of *P. aeruginosa* is able to export sodium in exchange for protons but cannot transport lithium [47], while Pa-NhaB can export sodium and lithium and does so preferentially at alkaline conditions [28]. In contrast to NhaP and NhaB, Pa-Mrp, also termed Sha in PA01 [26], is composed of multiple units (*mrpA’CDEFG*) and also facilitates sodium export and pH homeostasis at alkaline pH, with potential functions beyond cation transport. Interestingly, Pa-Mrp contributed to pathogenicity when assayed in mice, but had no influence in antibiotic resistance [26]. Overall, Mrp has also been shown to confer bile acid resistance when expressed in *Escherichia coli* [13] and *Vibrio cholerae* [13, 22], and played a role in nitrogen metabolism, cell motility and biofilm formation in *V. cholerae* [1]. In addition, putative *mrp* operons have been found in a large variety of pathogens and environmental strains that often survive in very extreme conditions [45].

In addition to growth in challenging environmental conditions, this study also investigates the contributions of these primary and secondary membrane transporters to biofilm formation and swarming motility. Swarming motility is the rapid, coordinated, and grouped movement of bacteria over a semi-solid surface [39]. Flagellar movement, which in *P. aeruginosa* enables swimming motility, also assists in swarming motility and is powered by the proton motive force [12, 42]. This force is generated by the transmembrane proton concentration gradient, created and maintained by primary or secondary proton pumps [12, 32]. Clearly, membrane cation transporters contribute to various cellular functions and a deeper understanding of these pumps is needed as they are opportunities for new drug targets [9, 10].

**Fig. 1:**
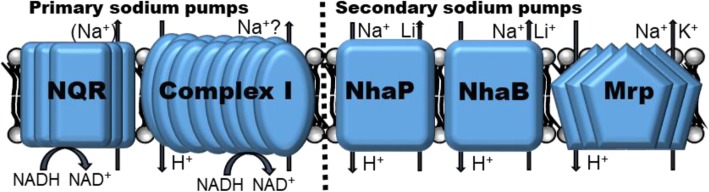
Primary and secondary sodium antiporter discussed in this article. NQR (NADH-ubiquinone oxidoreductase) is a major primary sodium pump in many species, but likely not *P. aeruginosa*. Complex I’s involvement as a primary sodium pump is questionable in *P. aeruginosa*. NhaP and NhaB are commonly transporting sodium and lithium and Mrp is a powerful transporter of sodium and potassium

The aims of this study are to assess the role of certain transporters of *P. aeruginosa* (Fig. 1) in different pH and sodium environments in regards to growth, biofilm formation, motility at pH 6.5, 7.5, and 8.5, and sodium concentrations up to 500 mM and also to assess their sensitivity to weak acids.

## Results

### Sodium and pH tolerance

**Fig. 2:**
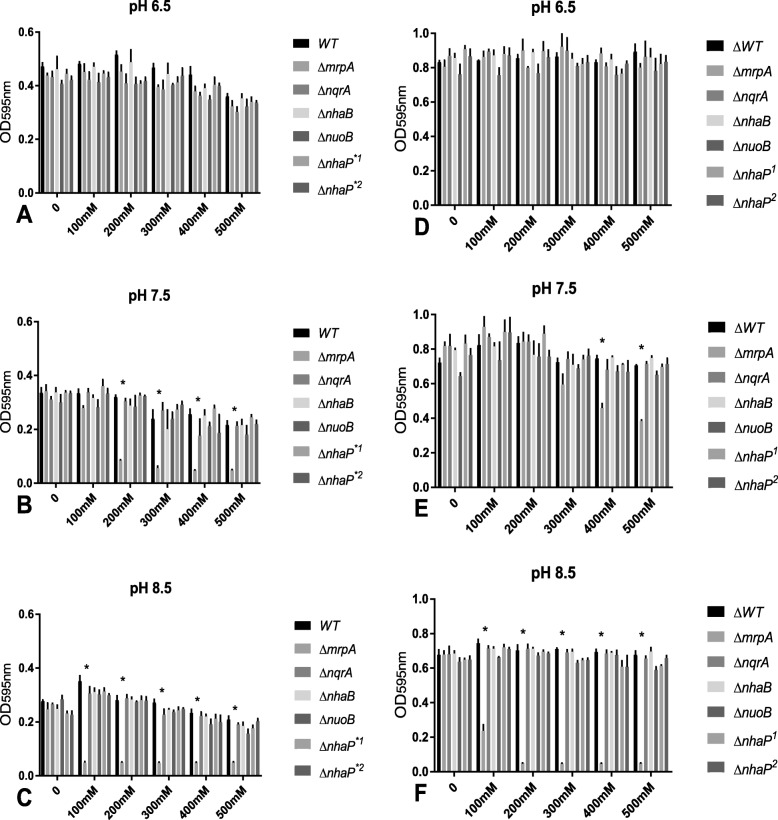
Comparison of *P. aeruginosa* mutants to wild-type strain (WT) grown with 0-500 mM added NaCl in early exponential phase (panels **a–c**) and early stationary phase (panels **d**–**f**). After 6 h of incubation at pH 6.5 (panel **a**), pH 7.5 (panel **b**), and pH 8.5 (panel **c**) and after 18 h of incubation at pH 6.5 (panel **d**), pH 7.5 (panel **e**), and pH 8.5 (panel **f**), optical density at 595 nm was measured. Columns indicate mean growth of three independent experiments of each two technical replicates, and error bars indicate standard error margin of the mean (SEM). Asterisks indicate statistically significant differences of the MrpA mutant to WT with the addition of ≥ 200 mM NaCl (panel **b**) or ≥ 400 mM NaCl (panel **d**) at pH 7.5 (panel **b**) and the addition of ≥ 100 mM NaCl at pH 8.5 (panels **c**, **f**) with *p* values smaller than 0.0001

All *P. aeruginosa* strains listed in Table 1 were grown at pH 6.5, 7.5, and 8.5 in LBB^-^ with no added sodium or added sodium ranging from 100 to 500 mM. While hourly measurements were taken, only time points 6 and 18 h are presented for better clarity of the graphs and the statistical analyses; this selection was arbitrary but the time points were representative of early exponential and early stationary phase, respectively. The full 24-h growth curves are available as supplemental data S1-3. Generally, strains grew best at pH 6.5 and worst at pH 8.5. While higher sodium conditions were disadvantageous for all strains in early exponential phase (Fig. 2a–c), those differences were less obvious at early stationary phase (Fig. 2d–f). In addition, variability between the biological replicates increased with increasing incubation length, which was likely related to exhaustion of the growth medium and were most severe at 24 h (Fig. 2d–f; S1-3). At acidic conditions, none of the mutations resulted in an increase in sodium sensitivity when compared to the wild-type strain, regardless of growth phase (Fig. 2a–d). At pH 7.5 and in exponential phase, the MrpA mutant was highly growth retarded in a sodium concentrations of 200 mM and higher (Fig. 2b, S2) and approached wild-type levels in the 200 and 300 mM condition after 18 h, but the strain was still highly growth deficient at sodium concentrations of 400 and 500 mM (Fig. 2e). At pH 8.5, growth was essentially arrested with the addition of ≥ 100 mM NaCl (Fig. 2c, f), with the exception that in 100 mM sodium, growth started to recover after 14 h, although the growth retardation continued to be statistically significant when compared to the wild-type strain (S3). The asterisks in the graphs indicate statistical significance (*p* < 0.0001; Fig. 2). It should be noted that the Complex I mutant often displayed less growth compare to the wild-type, but the differences were small and somewhat inconsistent.

**Table 1:**
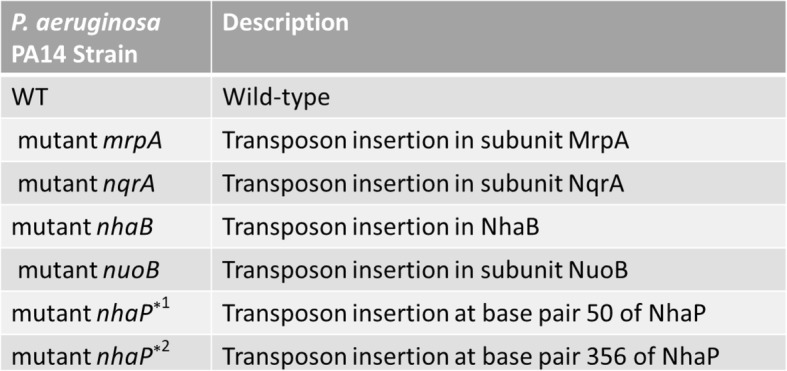
Strains used in this work. All strains were obtained from the *P. aeruginosa* PA14 transposon insertion library. The location of the insertion in the multi-subunit proteins Mrp, NQR and Nuo, is subunit A for Mrp and NQR, and subunit NuoB or 1 in Complex I. Two different mutants were available for NhaP, and labeled mutant nhaP*1 (insertion at base pair 50) and mutant nhaP*2 (insertion at base pair 356), respectively. Both NhaP mutants were included in our testing

### Biofilm assays

**Fig. 3:**
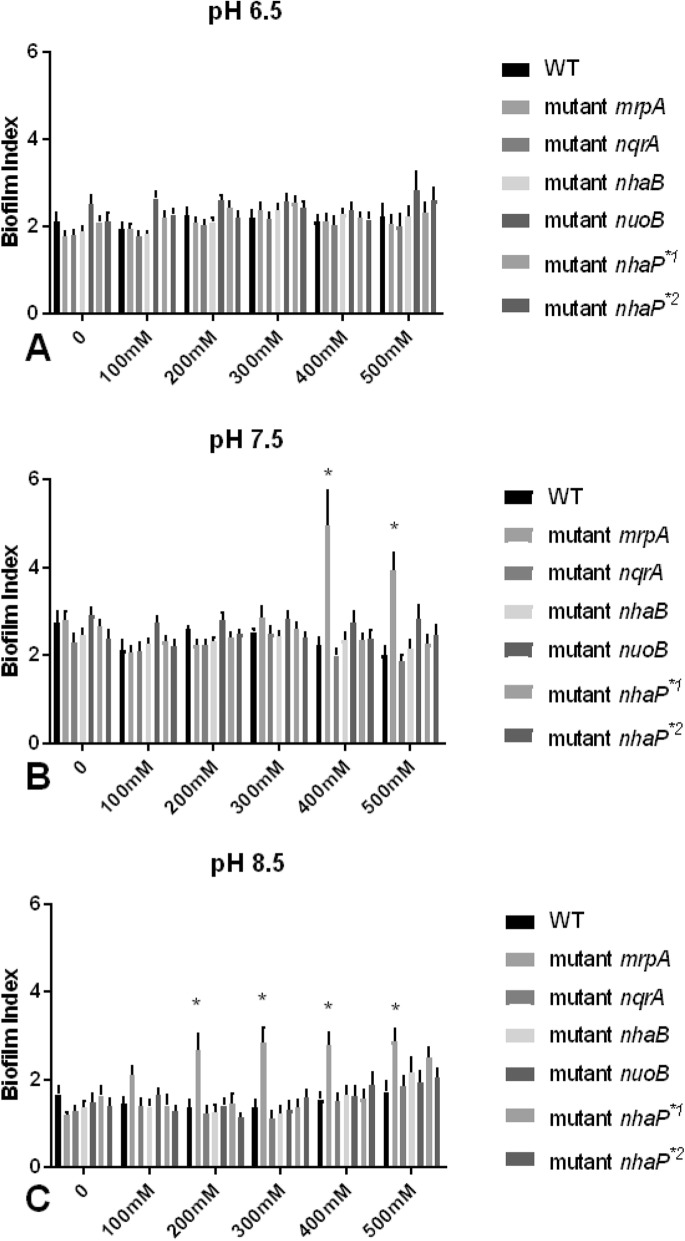
Biofilm index comparison of *P. aeruginosa* mutants to wild-type strain (WT) grown with 0–500 mM added NaCl in stationary phase. After 24 h of incubation at pH 6.5 (panel **a**), pH 7.5 (panel **b**), and pH 8.5 (panel **c**), optical density of the cultures in the wells was measured at 595 nm. Then, the biofilms in the wells was stained and the wells measured at 570 nm. The biofilm index was calculated as OD570/OD595 to account for growth differences. Columns indicate mean ratio of five independent experiments, and error bars indicate standard error margins of the mean (SEM). Asterisks indicate statistically significant differences in Biofilm Index of the MrpA mutant with the addition of ≥ 400 mM NaCl at pH 7.5 (panel **b**) or with the addition of ≥ 200 mM NaCl at pH 8.5 (panel **c**) to WT with *p* values equal or smaller than 0.001

To account for growth differences, a Biofilm Index was calculated as a ratio of OD570nm/595 nm (optical density at 570 nm–crystal violet staining divided by optical density of the culture in each respective well measured at 595 nm before the wash and staining steps). A higher index means more proportional biofilm production relative to growth. Biofilm formation at all sodium concentrations was overall the lowest at pH 8.5 (Fig. 3c). Within pH 8.5, highest Biofilm Index was calculated when 500 mM sodium chloride was added (Fig. 3c). Within the measurements at pH 6.5 and 7.5, slightly higher Biofilm Indexes were seen when no sodium was supplemented (pH 7.5) and at pH 6.5 with the addition 500 mM sodium chloride (Fig. 3a, b). Sodium additions ranging between 100 and 400 mM resulted in fairly similar Biofilm Indexes at pH 6.5 and 7.5 (Fig. 3a, b). At pH 6.5, there were no statistical significant differences between the wild-type and the mutant strains at any of the sodium conditions evaluated (Fig. 3a). The MrpA mutant displayed a severely heightened Biofilm Index in compare to the wild-type strain when 400 and 500 mM NaCl was added at pH 7.5, while at pH 8.5 the addition of ≥ 200 mM NaCl caused significantly higher Biofilm Indexes with a *p* value of < 0.001 (Fig. 3b, c).

### Swarming motility

**Fig. 4:**
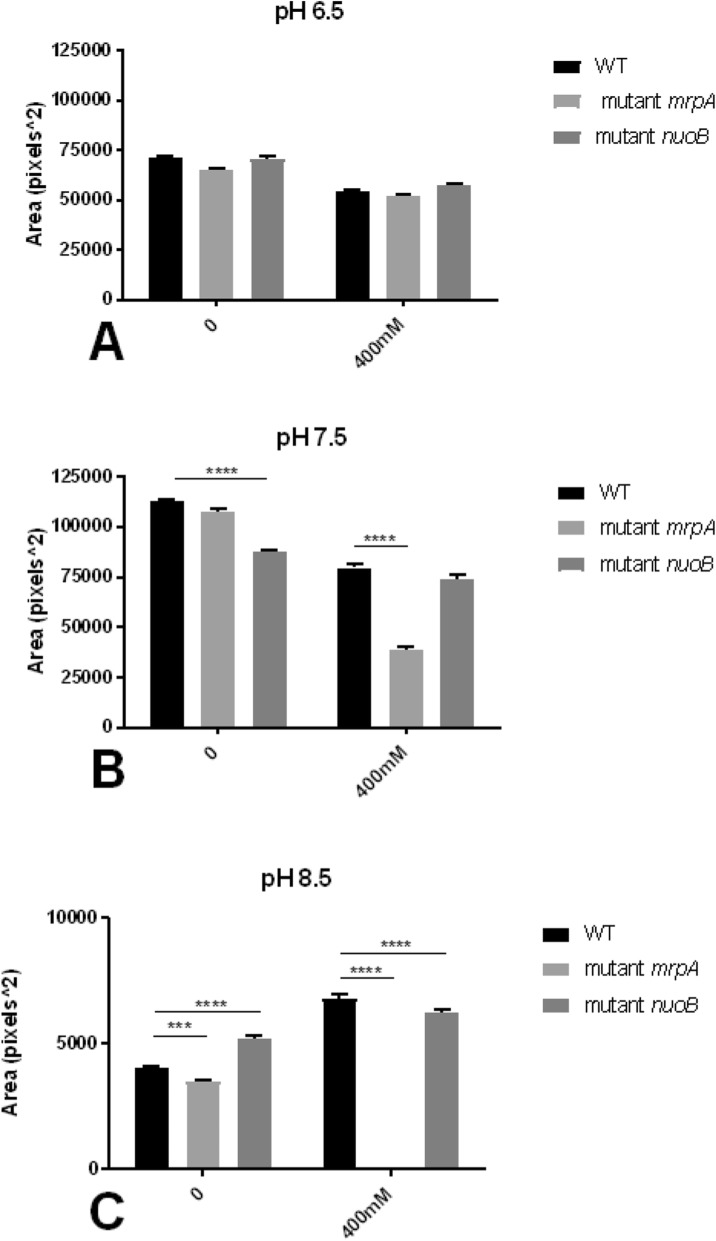
Swarming motility of the *P. aeruginosa* MrpA and Complex I mutants compared to the wild-type strain (WT). The swarming motility was accessed after 18 h on semi-solid agars of pH 6.5, 7.5, or 8.5 and no additional NaCl or when supplemented with 400 mM NaCl. Swarming motility was measured as area of growth with the software ImageJ and graphed as square-pixels. Representative photographs are shown in Fig. 5. No statistical differences were observed at acidic pH (panel **a**). At pH 7.5 and without added sodium, the Complex I mutant showed significantly reduced swarming motility in compare to WT (*p* < 0.0001) while the Mrp mutant but not the Complex mutant showed hypomotility when 400 mM NaCl was added (panel **b**). Very little swarming motility was detected at pH 8.5 (panel **c**), with significant hypermotility of the Complex I mutant when no sodium was added *p* < 0.0001), but reduced swarming of the Mrp mutant (*p* < 0.001). When at pH 8.5 (panel **c**), 400 mM NaCl was added, the Mrp mutant did not grow on the semi-solid agar (see Fig. 5), and the Complex I mutant revealed significantly reduced swarming motility in compare to WT (both *p* < 0.0001)

**Fig. 5:**
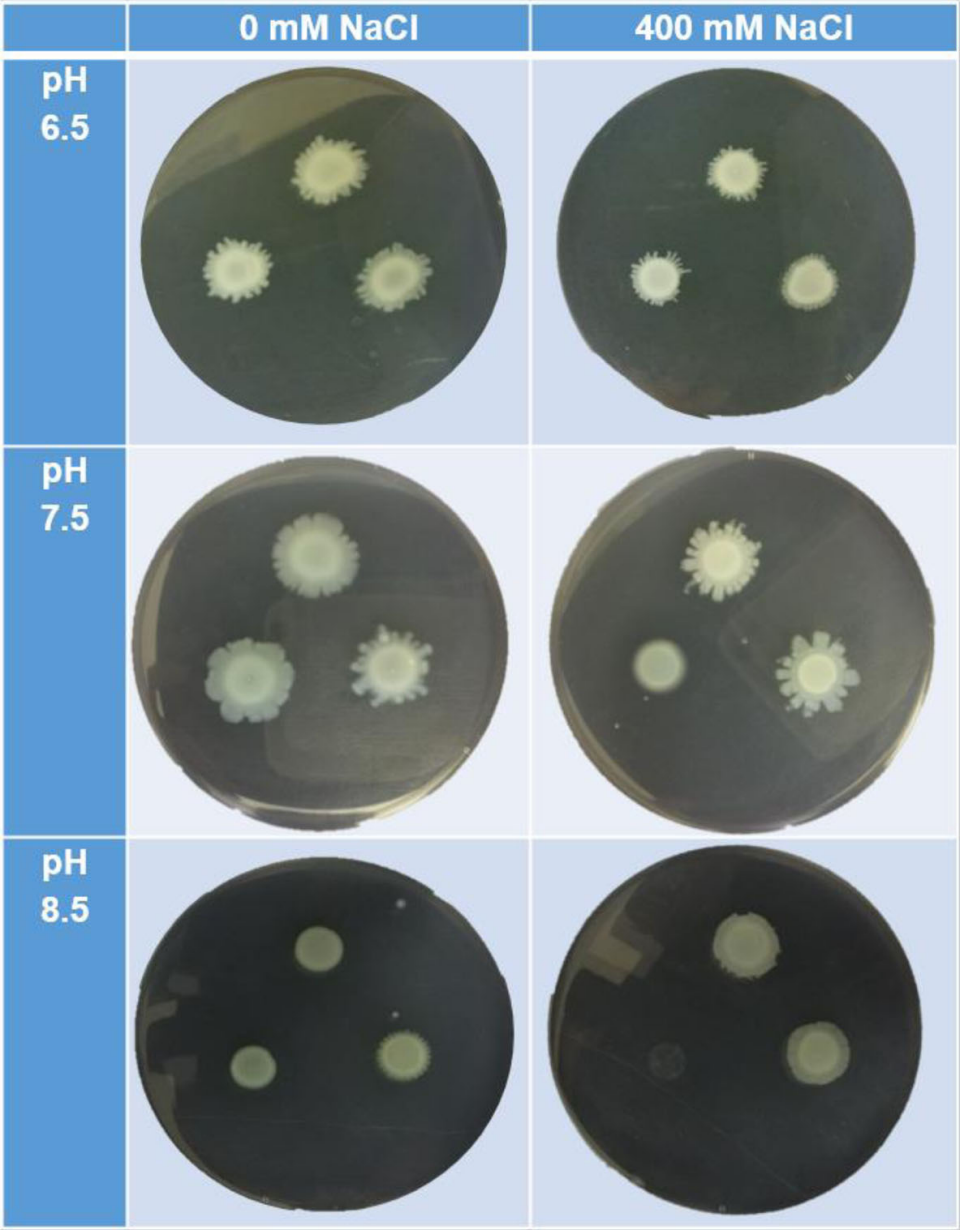
Swarming motility of the *P. aeruginosa* wild-type (WT), MrpA mutant and Complex I mutant strains. Five microliters of culture was applied onto the semi-solid agar of pH 6.5 (upper row), 7.5 (middle row), and 8.5 (lowest row), and without (0 mM NaCl, left column) or with 400 mM NaCl (right column) and grown for 18 h. In this figure are photographs of representative agar plates at each condition with three aliquots of bacterial culture arranged as top (WT), lower right (Complex I mutant), and lower left (MrpA mutant)

Swarming motility was assessed quantitatively by measuring the area of growth across the semi-solid surface after 18 h of incubation (Fig. 5). Preliminary experiments revealed that swarming motility was not affected by the mutations within NQR, NhaB, and NhaP (data not shown); thus, these experiments only compared the mutants of Complex I and Mrp to the wild-type strain (Fig. 5). Overall growth areas were the largest at pH 7.5 (Fig. 4b) and smallest at pH 8.5 (Fig. 4c). Sodium addition or mutation did not significantly affect the swarming motility at pH 6.5 (Fig. 4a). At pH 7.5 and no added sodium, the Complex I mutant showed significantly (*p* < 0.0001) less swarming thus covering less area on the agar plate (Fig. 4b). While when 400 mM NaCl was added, the Mrp mutant displayed less motility (*p* < 0.0001; Fig. 4b). At alkaline pH, both mutants were significantly different in compare to the wild-type (Fig. 4c). The Mrp mutant did not grow when 400 mM NaCl was added (*p* < 0.0001) (Fig. 5), and the Complex I mutant became hypermotile when no additional sodium was added (*p* < 0.0001) but also displayed significantly reduced swarming motility with the addition of 400 mM NaCl (*p* < 0.0001; Fig. 4c).

### Weak acid resistance

Our previously published work on Mrp in *V. cholerae* [1] identified susceptibility of the Mrp mutant to three weak acids: docusate, also known as dioctyl sulfosuccinate, *n*-lauroylsarcosine, and probenecid. Therefore, those weak acids were tested in this assay. Due to the structural similarities of Mrp to Complex I (reviewed in the discussion), we tested both mutants and compared them to the wild-type strain. Both mutants did not reveal any significant differences compared to the wild-type strain or the results were inconclusive, making the involvement of these enzymes in drug transport questionable (S4, 5, 6).

## Discussion

Sodium/proton antiporters are important membrane proteins that export sodium in exchange with protons for internal pH homeostasis and play roles in energy metabolism, nutrient acquisition, and more [35]. These transporter proteins can be found in a wide variety of eukaryotic and prokaryotic cells [35, 36, 49]. Loss of such proteins can have significant consequences to the cell thus making these proteins interesting drug target candidates. In this study, growth, biofilm formation, swarming motility, and resistance to weak acids under different environmental conditions were examined using transposon insertion mutants in *P. aeruginosa*. The primary transporters NQR and Complex I were studied, as well as the secondary antiporters Mrp, NhaB, and NhaP.

The loss of the NQR did not affect growth under challenging sodium and pH conditions, which confirms a recent study by Raba et al. [37] who suggested that in contrast to NQR in other species, such as *Vibrio* [3, 23, 27, 51], Pa-NQR is a proton pump with no affinity for sodium. In addition to that, our results suggest that loss of NQR does not affect pH homeostasis, biofilm formation, or swarming motility, which might be due to complementary proton pumping activity of Complex I. This hypothesis should be tested with a double deletion mutant of *nqr* and *nuo* in the future.

NhaB is not essential in *V. cholerae* as it has a supporting function and is, when lost, easily replaced by other sodium transporters, such as NhaA or NhaD, which are missing in *P. aeruginosa* [21]. Based on our results Pa-NhaB is unlikely a major contributor to sodium transport as its mutation did not interfere with growth, biofilm formation, or swarming motility at any of the tested conditions. In addition, it had been reported that when Pa-NhaB was expressed in everted membrane vesicles at pH 8.0 it showed a higher affinity to lithium than sodium [28]. This could suggest that Pa-NhaB has only a supportive function in sodium transport. To further investigate this theory, a triple deletion mutant strain lacking NhaA, NhaP, and Mrp should be constructed as these proteins can likely complement each other’s sodium transport in single mutants.

Pa-NhaP has also only been assayed when expressed in everted membrane vesicles of the antiporter-deficient *E. coli* strain KNabc, where Pa-NhaP was found to be a major sodium but not lithium transporter [28]. While we did not evaluate NhaP in regards to lithium, we tested two independent *P. aeruginosa* NhaP mutants in different pH and sodium conditions and did not observe an increase of sodium sensitivity compared to the wild-type strain. Also unaffected were biofilm formation and swarming motility. This lack of effect is likely due to a concerted effort of other sodium pumps (such as Mrp) in challenging environmental conditions and should be further investigated in the future.

When the MrpA subunit was mutated, neither growth nor biofilm formation or swarming was affected at pH 6.5, but significant changes occurred at pH 7.5 and particularly at pH 8.5. At pH 7.5 and early log phase, sodium concentrations of ≥ 200 mM led to a prolonged lag phase, while at pH 8.5, ≥ 200 mM NaCl resulted in a growth arrest from which the strain did not recover. An involvement of the *Pa*-*mrp* gene cluster (previously also designated *sha* [26];) as a major contributor to sodium transport at alkaline pH was previously demonstrated by the quenching method using everted membrane vesicles of the sodium sensitive *E. coli* TO114 which lacks three major Na^+^/H^+^ antiporters [41] and by disruption of *PA01*-*shaA* [26]. In our experiments, the Pa-Mrp mutant experienced a severely prolonged lag phase when exposed to high concentrations of sodium (400 and 500 mM NaCl) at pH 7.5 and a complete growth retardation for at least 24 h at pH 8.5 when ≥ 200 mM NaCl was added, but showed successful formation of biofilms under those conditions. This could be explained by altered physiological characteristics often found when planktonic bacteria become sessile, such as a vastly altered gene expression profiles and slowed growth rate intended to make the sessile form more resilient to adverse environmental conditions (suggested by [11]). When biofilm formation is high, motility/swarming activity is usually low; this phenomena is a basic function in bacterial physiology and is antagonistically regulated [5, 48].

Swarming is the rapid multicellular movement of bacteria across an > 0.3% agar surface along a nutrient gradient generated by the bacteria, and the movement is powered by a rotating flagella [24]. While in some bacteria, like *V. cholerae*, flagellar rotation is powered by sodium; this does not seem to be the case in *P. aeruginosa* [5, 12], which has a polar flagella driven by a dual set of motor proteins, MotA/B/Y and MotC/D [12]. Different to many other bacteria, swarming in *P. aeruginosa* seems to be more complex and in some capacity also supported by Type IV pili [25, 34]. Swarming is often defined as the surface-motility used to translocate cells to a more favorable environment [12]. It can therefore be assumed that lack of swarming in unfavorable environmental conditions might result in heightened biofilm formation; however, from our results we conclude that swarming, or lack-thereof, is a poor predictor of biofilm production in *P. aeruginosa*. Regardless of the generally favorable effects of sodium addition to growth of our tested strains at pH 7.5 and 8.5 (Fig. 2), the Complex I mutant showed significantly reduced swarming at pH 7.5 and no added sodium (Fig. 4b; *p* < 0.0001), where we would likely expect higher swarming to get the cells into a more sodium-rich environment. A possible explanation is that this transporter is a major contributor to the protein motive force and thus provides the power necessary for flagella rotation. However, generally, this mutation did not alter any other phenotypes tested in this study, suggesting that other proteins collaborate with Complex I to enable normal phenotypes. In contrast at pH 8.5, the Complex I mutant revealed an increased swarming phenotype at the unfavorable sodium-depleted condition and reduced swarming in sodium-rich conditions, which supports the translocation to more favorable conditions idea (Fig. 4c; *p* < 0.0001). Clearly, future studies are required to better understand these phenotypes.

The Mrp mutant showed significantly reduced swarming motility compared to the wild-type strain at pH 7.5 and 400 mM added sodium (Fig. 4b; *p* < 0.0001). However, at these environmental conditions, the strain’s growth was also significantly slowed and reduced compared to the wild-type strain (Fig. 2e), confirming Mrp’s involvement in sodium transport. However, this reduced growth could also explain the quantitatively reduced, sodium-independent swarming motility (Fig. 4b). Overall, the Mrp protein becomes most relevant when sodium levels in the environment are elevated (≥ 200 mM) and pH is 7.5, and it becomes essential at pH 8.5 in the presence sodium. In addition at those conditions (≥ pH 7.5 and ≥ 200 mM NaCl), lack of a functional Mrp protein complex resulted in higher biofilm formation, even though at pH 8.5 no growth and thus no swarming motility were evident. In summary, Mrp is clearly important to *P. aeruginosa*’s survival in challenging environments.

Mrp and Complex I seem to have some evolutionary relationship based on the homology between MrpC and NuoK [31], MrpA and NuoL, and MrpD and NuoMN [33]. Interestingly, the NuoL subunit has been suggested to transport sodium in *E. coli* [17, 22, 43]. While not reported as statistically significant in this work due to our stringent *p* value threshold and the randomness of the occurrences, a few differences between the wild-type and the NuoB mutant might be notable, for example at pH 8.5, the growth of the NuoB mutant was slightly reduced when 500 mM NaCl was added (*p* = 0.0165), or slightly elevated Biofilm Indexes were seen at pH 6.5 and 100 mM NaCl (*p* = 0.0246), as well as at pH 7.5 and 500 mM NaCl (*p* = 0.0323).

## Conclusion

Antiporters are membrane proteins that facilitate a variety of tasks to enable survival in quickly changing, and often challenging environmental conditions. Such tasks include intracellular pH homeostasis and cation as well as nutrient transport across the cellular membrane. In addition, antiporters often also contribute to flagellar movements. We have found that in *P. aeruginosa* the antiporters NQR, NhaB, and NhaP have only subordinate roles in maintaining normal cell physiology when exposed to high sodium and/or high pH environments. In contrast, Complex I, while unlikely essential for sodium transport, seems to be involved in swarming ability, and most importantly, Mrp is indispensable for growth in challenging sodium and pH conditions.

## Methods

All strains were originating from a *P. aeruginosa* strain PA14 transposon insertion library [30]. Due to different locations of the transposon insertion in NhaP, two different mutants of NhaP were evaluated (Table 1). Strains were isolated from the frozen stocks by streaking them on Luria-Berani (LB) agar and incubating them overnight at 37 °C. Fresh single colonies were cultured in LB Lennox broth (5 g per L of NaCl) in a roller drum or table top shaker at 7×*g* at 37 °C overnight. For the growth curves, buffered LBB^−^ (Luria broth without any additional sodium chloride; 10 g per L peptone, 5 g per L yeast extract, 60 mM BIS-TRIS propane) was used and the residual amount of sodium in our LBB^−^ was 13–19 mM Na^+^.

### Sodium and pH tolerance growth assays

All wells of a 96-well flat-bottom cell culture plate (Greiner Bio-one) were filled with 140 μl of LBB^−^ media of pH 6.5 and increasing sodium concentrations of 0, 100, 200, 300, 400, and 500 mM in technical duplicates throughout the 12 well columns. Overnight cultures of each strain were pelleted by centrifugation at 3800×*g* for 5 min, washed with LBB^−^, and re-suspended to an optical density of 0.1 measured by wavelength 600 nm (OD_600_). Twenty microliters of each culture was transferred to their respective wells, while control wells received 20 μl of sterile broth. All wells were then covered with 50 μl of mineral oil to prevent evaporation without affecting growth [7] and incubated at 37 °C and medium intensity shaking in a BioTek SynergyMx microplate reader for 24 h. The OD_595_ was measured hourly for during that time. The plate setup and the measurements were repeated for pH 7.5 and 8.5 and a total of three biological replicates per pH.

### Biofilm assays

For this assay, wells of a 96-well flat-bottom cell culture plate (Greiner Bio-one) were filled with 180 μl of pH and sodium adjusted LBB^−^ media. Again, the tested pH values were 6.5, 7.5, and 8.5, and sodium concentrations ranged from zero to 500 mM NaCl added. Cultures were re-suspended to an OD_600_ of 0.1 and 20 μl was added to each respective well in duplicates for each condition. Using an iMark plate reader (BioRad), the optical density at OD_595_ was recorded for each well after a 24-h incubation at 37 °C (no shaking). Then, the media was discarded and the plates were washed three times with deionized water and left to dry at room temperature. One hundred microliters of an 0.1% (w/v) crystal violet stain was added to each well and shaken at 7×*g* and 37 °C in an orbital shaker (Thermo Scientific MaxQ 4000) for 30 min. After staining, the plates were washed three times with deionized water and left to dry at room temperature. Then, 200 μl of 95% ethanol was added to the wells to solubilize the stained biofilm and consequently incubated for 15 min at room temperature. Optical density at 570 nm was measured in the iMark plate reader to assess the biofilm formation, and the biofilm ratio (OD_570_/OD_595_) was calculated to account for growth deficiencies [1]. These experiments were independently repeated five times for each pH.

### Swarming motility

LBB^-^ semi-solid agar plates (0.5% *w*/*v* agar) were adjusted to pH 6.5, 7.5, or 8.5, and supplemented with either 0 or 400 mM sodium chloride. Overnight cultures of each strain were pelleted in a table top centrifuge at 3800×*g* for 5 min and re-suspended in LBB^-^ media of pH 6.5, 7.5, or 8.5 to an OD_600_ of 0.3. For each strain, 5 μl of culture was delivered to the surface of the agar. The cultures were left to dry for 15 min at room temperature on the bench before being moved to the incubator where they were incubated at 37 °C for 18 h. All agar plates were photographed with 16 megapixel digital camera and the photographs analyzed using the freely available software ImageJ [38] and the ImageJ plugin Simple Interactive Object Extraction (SIOX) [14]. The experiment was independently repeated eight times, with two technical replicates per experiment.

### Weak acid resistance

Since most statistically significant differences in growth and swarming motility were limited to the mutants *mrpA* and *nuoB*, and only these two mutants were submitted to the weak acid resistance evaluation. Overnight cultures of the wild-type strain and the MrpA and NuoB mutant were pelleted in a tabletop centrifuge at 3800*×g* for 5 min and re-suspended with fresh LB media (pH ± 7.0) to an OD_600_ of 0.1. Wells of a 96-well flat-bottom cell culture plate (Greiner Bio-one) were filled with 120 μl LB media, and 20 μl of each cell culture was added to each respective well. The weak acids tested were docusate, *n*-lauroyl sarcosine, and probenecid. Probenecid was dissolved into dimethyl sulfoxide (DMSO) prior to use. Docusate was tested as six two-fold serial dilutions from 2.5 mg to 0.039 mg per mL LB broth, and *n*-lauroylsarcosine and probenecid were tested at 10-fold higher concentrations than docusate. Wells that did not receive any weak acids served as no-drug controls. The assay with probenecid contained additional no-drug controls where the strains were grown with DMSO. The wells containing *n*-lauroylsarcosine and probenecid were overlaid with 50 μl mineral oil, and the plates were incubated at 37 °C and shaking at medium intensity. Growth measurements were recorded at OD_595_ every hour for 24 h. Plates with docusate were covered with lids and wrapped in parafilm and incubated at 37 °C and shaking at 7×*g* in an orbital shaker for 24 h. Growth measurements were taken every 4-h using an iMark plate reader (BioRad). Each strain was tested in technical duplicates, and the experiment was independently repeated eight times for docusate, three times for probenecid, and six times for *n*-lauroylsarcosine.

### Statistical analysis

For all statistical analyses, GraphPad Prism version 7.0 for Windows (GraphPad Software, La Jolla California USA) was used. A two-way ANOVA and Dunnett’s multiple-comparison tests were employed on all data sets that were previously confirmed to be normally distributed by D’Agostino-Pearson normality test. All results were compared to the wild-type, and *p* ≤ 0.001 was considered statistically significant unless otherwise noted.

### Abbreviated summary

*Pseudomonas aeruginosa’*s secondary sodium pump Mrp is crucial for survival, growth, biofilm formation, and swarming motility of the organism in challenging environmental conditions (high pH and sodium). In contrast, lack of the primary sodium pump Complex I had some consistent impact on *P. aeruginosa*’s growth, but those changes were independent of pH and sodium concentration. Additionally, the primary sodium pump NQR and the secondary sodium pumps NhP and NhaB played only minimal or insignificant roles.

## Data Availability

The datasets used and/or analyzed during the current study are available from the corresponding author on reasonable request.

## Abbreviations

*w/v*: weight per volume
